# Prevalence and correlates of carotid plaque in a mixed HIV-serostatus cohort in Uganda

**DOI:** 10.1186/s12872-021-02416-5

**Published:** 2021-12-15

**Authors:** Prossy Bibangambah, Linda C. Hemphill, Moses Acan, Alexander C. Tsai, Ruth N. Sentongo, June-Ho Kim, Isabelle T. Yang, Mark J. Siedner, Samson Okello

**Affiliations:** 1 Department of Radiology, Mbarara University of Science and Technology, P.O. Box, 1410 Mbarara, Uganda; 2 Massachusetts General Hospital, Boston, MA USA; 3 Harvard Medical School, Boston, MA USA; 4 Geisel School of Medicine at Dartmouth, Hanover, NH USA

**Keywords:** HIV, Carotid intima media thickness, Carotid plaque, Atherosclerosis, Cardiovascular disease

## Abstract

**Background:**

The extent to which the risk of atherosclerotic cardiovascular disease (ACVD) is increased among people living with HIV (PLWH) in sub-Saharan Africa remains unknown.

**Setting:**

Cross-sectional analysis nested within the Ugandan Noncommunicable Diseases and Aging Cohort, including PLWH in rural Uganda > 40 years taking antiretroviral therapy (ART) for at least 3 years, and a population-based control group of HIV-uninfected age- and sex-matched persons.

**Methods:**

We conducted carotid ultrasonography and collected ACVD risk factor data. Our outcome of interest was carotid plaque, defined as > 1.5 mm thickness from the intima-lumen interface to the media-adventitia interface. We fit multivariable logistic regression models to estimate correlates of carotid plaque including HIV-specific and traditional cardiovascular risk factors.

**Results:**

We enrolled 155 (50.2%) PLWH and 154 (49.8%) HIV-uninfected comparators, with a mean age of  51.4 years. Among PLWH, the median CD4 count was 433 cells/mm^3^ and 97.4% were virologically suppressed. Carotid plaque prevalence was higher among PLWH (8.4% vs 3.3%). HIV infection (aOR 3.90; 95% CI 1.12–13.60) and current smokers (aOR 6.60; 95% CI 1.22–35.80) had higher odds of carotid plaque, whereas moderate (aOR 0.13, 95% CI 0.01–1.55) and vigorous intensity of physical activity (aOR 0.34, 95% CI 0.07–1.52) were associated with decreased odds of carotid plaque.

**Conclusion:**

In rural Uganda, PLWH have higher prevalence of carotid plaque compared to age- and sex-matched HIV-uninfected comparators. Future work should explore how biomedical and lifestyle modifications might reduce atherosclerotic burden among PLWH in the region.

## Background

There are few data on the epidemiology of atherosclerosis among PLWH in sub-Saharan Africa, which is home to at least 70% of the global burden of HIV [1]. Most of the data available from this region has focused primarily on the epidemiology of traditional risk factors of atherosclerosis or its clinical manifestations [2–5]. The few studies directly measuring atherosclerosis among PLWH in sub-Saharan Africa have shown conflicting results with some studies describing an increased burden of atherosclerosis among PLWH [6], others no difference [7] and others a reversal [8].

We sought to respond to this gap by estimating the prevalence of carotid plaque in a Ugandan cohort of PLWH and age- and sex-matched HIV-uninfected comparators. To do so, we conducted carotid ultrasound to detect carotid plaque, a validated surrogate marker and predictor of atherosclerotic CVD risk [9, 10]. Previous studies have shown that there was no difference in carotid intima media thickness (cIMT), another predictor of atherosclerotic CVD risk, between PLWH and uninfected comparators [11, 12]. Other cohorts, however, like the MultiCenter AIDS Cohort Stuudy (MACS) in the US have shown that while there was no difference in cIMT, there was a difference in carotid plaque [13] and this remains unstudied in Uganda. We hypothesized that the prevalence of carotid plaque would be higher among the PLWH compared with their HIV-uninfected comparators.

## Methods

### Study population

We conducted a cross-sectional within the Ugandan Noncommunicable Diseases and Aging Cohort (UGANDAC) study (NCT02445079) [14, 15]. Briefly, UGANDAC was a prospective cohort of ambulatory PLWH aged at least 40 years on ART for at least 3 years, and a matched population-based comparator group of uninfected persons. The PLWH were enrolled as a convenience sample of HIV-infected persons receiving care at the Mbarara Regional Referral Hospital HIV clinic. We then used a complete population census study in a nearby cluster of villages [16] to identify and conduct home-based enrollment of sex-matched and age-similar (by quartile of the HIV-infected sub-group) HIV-uninfected comparators, who underwent confirmatory HIV testing prior to enrollment.

### Carotid ultrasonography

All study participants underwent carotid ultrasonography at the enrollment visit. Two operators (JHK and PB) trained in carotid ultrasonography at the University of Wisconsin, USA [17, 18] and blinded to participant information individually collected images using a 13-6 MHz linear transducer (Sonosite M-Turbo, Sonosite, Bothell, Washington, USA). We used a standardized carotid ultrasound scanning protocol to collect bilateral common carotid artery images in a longitudinal view from the anterior, lateral, and posterior positions [17]. We used validated semi-automated software (SonoCalc 5.0, SonoSite) [19] to measure carotid plaque, which was defined as a focal thickness of 1.5 mm or more protruding into the arterial lumen from the media-adventitia interface to the intima-lumen interface of the common carotid artery [17, 20]. A board-certified cardiologist (LH) reviewed image interpretation for all plaque measurements.

### Covariate data collection

The data used in this analysis were collected from participants at their enrollment study visit. Participants completed questionnaires on sociodemographic characteristics, physical activity, smoking history and history of medications including statin use. We measured weight using standardized scales (seca 762, Hanover, USA) and height using roll-up measuring stadiometers (seca 206, Hanover, USA).

We also collected resting blood pressure using digital sphygmomanometers (Omron Healthcare Inc., Bannockburn, USA). We collected blood samples for hemoglobin A1c testing (Siemens Vantage, Siemens Healthcare Diagnostics, Tarrytown, NY, USA) and lipid profile assessments (cryopreserved specimens at the Laboratory for Clinical Biochemistry Research at the University of Vermont, USA). Among PLWH, we abstracted CD4 + T-cell count and HIV-1 RNA viral load data from medical records.

### Study definitions and statistical analysis

We calculated the body mass index (BMI) as weight (in kilograms) divided by the square of height (in meters) and categorized it as underweight/low if less than 18.5 kg/m^2^, normal if between 18.5-25 kg/m^2^, overweight if between 25–29.9 kg/m^2^, and obese if 30 kg/m^2^ or more [21]. Self-reported smoking status was categorized as current, former (someone who quit smoking more than twelve months prior to enrollment), and never smoker. We defined physical activity using the physical activity categories determined by the International Physical Activity Questionnaire Protocol [22]. We defined virologic suppression as having less than 1000 copies of HIV per milliliter of blood [23].

### Statistical analysis

We used descriptive statistics to describe clinical and demographic characteristics for the total cohort and stratified by HIV serostatus. We graphically depicted prevalence of carotid plaque, stratified by HIV status and age (categorized as 40–49, 50–59 and 60 or more years). To estimate unadjusted and adjusted correlates of carotid plaque, we fit univariable and multivariable logistic regression models, including HIV serostatus as the primary explanatory variable of interest, and additional CVD risk factors, including sex, age, mean diastolic blood pressure, mean systolic blood pressure, glycated hemoglobin (%HbA1c), physical activity, total cholesterol, smoking status and BMI. A p-value of < 0.05 was considered statistically significant.

We conducted data analysis with Stata version 15.0 (StataCorp, College Station, Texas) and graphically presented prevalence of carotid plaque using Microsoft Excel 2016 (Microsoft Corporation, Washington).

### Ethical consideration

The UGANDAC study procedures were approved by the human subjects review boards at the Mbarara University of Science and Technology (Ref. 06/04-14), Partners Healthcare (Ref. 2014P001928/MGH), and the Uganda National Council for Science and Technology (Ref. HS1689). All participants provided written informed consent.

## Results

We enrolled 309 study participants: 155 (50.2%) were PLWH and 154 (49.8%) were HIV-uninfected. As expected based on the study design, mean age and sex were similar by HIV serostatus (Table 1). Among PLWH, the median CD4 at enrollment count was 433 (IQR, 336,559) cells/ml and 97.4% were virologically suppressed. About 64% were taking a nevirapine (NVP)-based ART regimen, 27.1% taking an efavirenz (EFV)-based ART, and 9.0% taking Protease inhibitors (Table 1). There was no statin use amongst study participants. Compared with the uninfected comparators, PLWH had lower systolic blood pressure (112.5 mmHg vs 117.5 mmHg; *P* = 0.015) and lower diastolic blood pressure (69 mmHg vs 77 mmHg; *P* < 0.001), and were less likely to be current smokers (5.8% [9/155] vs 20.8% [32/154]; *P* < 0.001).

**Table 1:** Study participant characteristics at Enrollment into a Mixed HIV-Serostatus Cohort in Uganda

Characteristic	Overall (n = 309)	HIV-negative (n = 154)	PLWH (n = 155)	*P*-value*
Age, years (mean, SD)	51.4 (7.2)	51.5 (7.7)	51.2 (6.6)	0.695
Female (n, %)	151 (48.9)	77 (50.0)	74 (47.7)	0.691
Smoking status (n, %)				< 0.001
Never-smoker	163 (52.8)	72 (46.8)	91 (58.7)	
Former smoker	105 (34.0)	50 (32.5)	55 (35.5)	
Current smoker	41 (13.3)	32 (20.8)	9 (5.8)	
Physical activity^β^ (n, %)				0.003
Low intensity	16 (5.2)	3 (2.0)	13 (8.4)	
Moderate intensity	42 (13.6)	15 (9.7)	27 (17.4)	
Vigorous intensity	251 (81.2)	136 (88.3)	115 (74.2)	
Body mass index (n, %)				0.056
Underweight (< 18.5 kg/m^2^)	40 (12.9)	27 (17.5)	13 (8.4)	
Normal (18.5- ≤ 25 kg/m^2^)	189 (61.2)	90 (58.4)	99 (63.9)	
Overweight/Obese (> 25 kg/m^2^)	80 (25.9)	37 (24.0)	43 (27.7)	
Hemoglobin A1c, % (mean, SD)	5.5 (1.0)	5.6 (0.8)	5.4 (1.1)	0.287
Total cholesterol, mg/dL (mean, SD)	160.1 (35.9)	159.5 (33.2)	160.6 (38.4)	0.788
Mean systolic BP, mmHg (median, IQR)	114.5 (105.5, 126.5)	117.5 (108, 131.5)	112.5 (100, 120)	0.015
Mean diastolic BP, mmHg (median, IQR)	73 (66, 81.5)	77 (68.5, 83.5)	69 (63.5, 79.0)	< 0.001
Virologic suppression^Ω^ at enrollment (n, %)	N/A	N/A	150 (97.4)	
Nadir CD4 count, cells/µ (median, IQR)	N/A	N/A	118 (74, 183)	
CD4 count at enrollment, cells/µ (median, IQR)	N/A	N/A	433 (336, 559)	
ART Regimen (n, %)	N/A	N/A		
NVP-based	N/A	N/A	99 (63.9)	
EFV-based	N/A	N/A	42 (27.1)	
PI-based	N/A	N/A	14 (9.0)	

*IQR* interquartile range; *SD* standard deviation; *ART regimen* antiretroviral therapy regimen; *AZT* zidovudine; *3TC* lamivudine; *NVP* nevirapine; *EFV* efavirenze; *TDF* tenofovir; *PI* protease inhibitor; *N/A* not applicable

^*^P-values comparing the PLWH with the HIV-uninfected were calculated using chi-squared testing for categorical variables and *t* tests for normally distributed continuous variables

^β^Physical activity categories were determined by the International Physical Activity Questionnaire (IPAQ) scoring protocol

^Ω^Virologic suppression defined as having less than 1000 copies of HIV per milliliter of blood

**Fig. 1:**
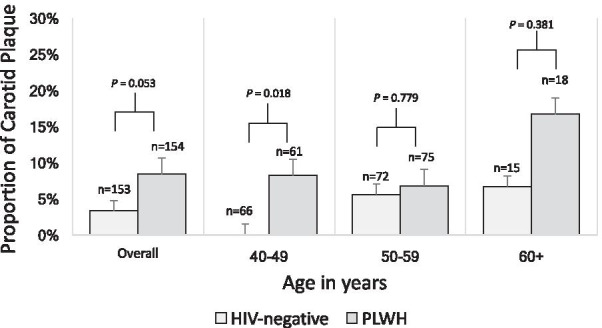
Plaque prevalence by age (years) and serostatus in a Mixed HIV-serostatus cohort in Uganda

The prevalence of carotid plaque was 5.8% (95% confidence interval [CI] 3.7–9.1) in the total cohort. PLWH had a higher prevalence than HIV uninfected comparators (8.4% vs 3.3%). The prevalence of carotid plaque was highest among those aged 60 years and over, and tended to be higher among PLWH in all age groups but these estimates were not statistically significant (Fig. 1).

In multivariable regression models (Table 2), HIV-infection (aOR 3.90; 95% CI 1.12–13.60, *P* = 0.033) and current smoking status (aOR 6.60 for current versus never smokers; 95% CI 1.22–35.80, *P* = 0.029) were significantly associated with higher odds of carotid plaque. We also found that moderate (aOR 0.13, 95% CI 0.01–1.55, *P* = 0.107) and vigorous physical activity (aOR 0.34, 95% CI 0.07–1.52, *P* = 0.156) were associated with decreased odds of carotid plaque, but these estimated associations were not statistically significant.

**Table 2:** Correlates of Carotid Plaque in a Mixed HIV-Serostatus Cohort in Uganda

Characteristic	Unadjusted model		Adjusted model	
	Odds Ratio (95% CI)	*P*-value	Odds Ratio (95% CI)	*P*-value
HIV Serostatus				
HIV-uninfected	REF	REF	REF	REF
PLWH	2.73 (0.95, 7.85)	0.063	3.90 (1.12, 13.60)	0.033
Age (each year)	1.04 (0.99, 1.10)	0.146	1.04 (0.97, 1.12)	0.268
Mean systolic BP (each mmHg increase)	1.02 (0.99, 1.04)	0.183	1.01 (0.96, 1.06)	0.831
Mean diastolic BP (each mmHg increase)	1.02 (0.00, 0.39)	0.446	1.04 (0.95, 1.13)	0.425
Physical Activity				
Low	REF	REF	REF	REF
Moderate	0.11 (0.10, 1.11)	0.061	0.13 (0.01, 1.55)	0.107
Vigorous	0.26 (0.07, 1.00)	0.051	0.34 (0.07, 1.52)	0.156
Smoking status				
Never-smoker	REF	REF	REF	REF
Current smoker	2.83 (0.76, 10.54)	0.121	6.60 (1.22, 35.80)	0.029
Former smoker	2.16 (0.73, 6.41)	0.166	2.37 (0.68, 8.30)	0.176
Female	0.65 (0.25, 1.72)	0.386	0.90 (0.27, 2.92)	0.855
Body mass index, kg/m^2^				
Normal	REF	REF	REF	REF
Underweight	0.35 (0.04, 2.73)	0.315	0.26 (0.03, 2.25)	0.221
Overweight/Obese	0.71 (0.23, 2.26)	0.564	0.85 (0.20, 3.58)	0.823
Total cholesterol (each mg/dL increase)	0.99 (0.98, 1.01)	0.583	0.99 (0.98, 1.01)	0.513
Hemoglobin A1c	0.67 (0.28, 1.57)	0.347	0.77 (0.31, 1.88)	0.564

## Discussion

In this mixed HIV cohort in rural Uganda, we found the prevalence of carotid plaque was higher among PLWH compared with their HIV-uninfected counterparts. These findings align with previous studies, which have reported a higher prevalence of carotid plaque among PLWH compared with HIV-uninfected comparators [7, 13, 24–27]. For instance, a combined analsysis from the Women’s Interagency HIV Study and Multicenter AIDS Cohort Study reported that PLWH had a 61% greater risk of new focal carotid artery plaque formation over seven years compared with uninfected individuals, regardless of baseline vascular phenotype and after adjusting for cardiometabolic risk factors [13]. Importantly, the relationship between HIV and the presence and formation of plaque in this literature has been consistently observed even after adjustment for other correlates of atherosclerosis. While the mechanistic details will require further study, the increased burden of carotid plaque among PLWH has been hypothesized to be mediated by persistent immune activation and inflammation that occurs in HIV infection [14, 28, 29].

Our reported prevalence of carotid plaque is lower than that found in other studies done within sub-Saharan Africa [30]. The difference in these findings could be due to the study populations, for example, an inclusion criteria from a similar study in Malawi was presentation of an acute stroke-like syndrome, whereas our study population was comprised of asymptomatic participants in ambulatory care. We found higher odds of carotid plaque among those who were current smokers and a lower odds among those with moderate or vigorous physical activity. These data corroborate studies that have described physical inactivity and smoking as independent predictors of carotid plaque development [31–34]. Although we found that the prevalence of carotid plaque was higher among those aged 60 and older, we did not find significant associations between age and carotid plaque, possibly due to the relatively narrow age distribution in our study. This finding differs from other studies which have found that age has been associated with risk of carotid plaque [27].

Notably, we previously demonstrated no association in this cohort between HIV serostatus and cIMT thickness or progression [11]. While both cIMT and carotid plaque are considered predictors of atherosclerotic disease events, they vary in pathophysiology whereby cIMT represents intimal medial thickening with very early atherosclerotic changes in the intima but also, potentially, smooth muscle hypertrophy and/or hyperplasia in the media which is more related to hypertension. Alternatively, carotid plaque is predominantly intimal thickening with foam cells, smooth muscle cells, macrophages, a lipid core, and a fibrous cap depending on the age of the plaque [10]. This variation in pathophysiology could explain the difference in findings between these two modalities. It is also in keeping with studies from the United States, for example the MACS study, that similarly demonstrated greater incidence of carotid plaques in PLWH compared to HIV-uninfected, comparators but not with cIMT [13].

There is scarcity of policies that would support the integration of CVD care and coverage amongst PLWH in the region. The primary care guidelines for HIV/AIDS have been largely successful, yet integrated care for screening, diagnosis and management of CVD in this population is still lacking. Our data responds to the gap in the knowledge of the epidemiology of CVD risk that could stand as a foundation for further studies that develop the policies that could improve upon this integrated care. This would advance the healthcare delivery system, not only in rural Uganda, but also sub-Saharan Africa.

Our study was strengthened by the use of a well-characterised study cohort. However, we are unable to extrapolate beyond correlations or comment on progression or incidence of plaque due to our cross-sectional design. There could also be residual or unmeasured confounding from factors not included in our model that account for the increased prevalence seen among PLWH. This study was also limited by dichotomous measure of plaque. Prior studies have shown that continuous measures of plaque (e.g. carotid plaque area) are also strong predictors of CVD and can be used in targeting preventive therapy [35–37]. Finally, our study might be under-powered to demonstrate smaller effect sizes.

## Conclusion

In conclusion, we found a higher odds of carotid plaque among PLWH and smokers in rural southwestern Uganda. These results suggest a combination of HIV-specific and traditional risk factors might contribute to atherosclerosis in this population. Future work should explore the mechanisms underlying this observation, and determine whether lifestyle factors or other targeted interventions might reduce atherosclerotic burden among PLWH in the region.

## Data Availability

The datasets used and/or analysed during the current study are available from the corresponding author on reasonable request.

## Abbreviations

BMI: Body mass index
CI: Confidence Interval
CIMT: Carotid intima media thickness
CVD: Cardiovascular disease
HBA1c: Glycated hemoglobin
MACS: Multicenter AIDS Cohort Study
PLWH: People living with HIV
aOR: Adjusted odds ratio
UGANDAC: Ugandan Noncommunicable Diseases and Aging Cohort study
